# Stroke-Associated Pneumonia and Impaired Functional Recovery After Stroke: The Role of Nutritional-Inflammatory Factors

**DOI:** 10.3390/arm94020023

**Published:** 2026-04-06

**Authors:** Rongjian Feng, Chonggui Jiang, Yan Yang, Mao Su, Meng Qin, Quan Wei

**Affiliations:** 1 Rehabilitation Medicine Center and Institute of Rehabilitation Medicine, Center for Preclinical Safety Evaluation of Drugs, West China Hospital, Sichuan University, Chengdu 610041, China; fengrongjian0504@gmail.com; 2 Key Laboratory of Rehabilitation Medicine in Sichuan Province, West China Hospital, Sichuan University, Chengdu 610041, China; 3 Department of Rehabilitation Medicine, Sichuan Academy of Medical Sciences & Sichuan Provincial People’s Hospital, School of Medicine, University of Electronic Science and Technology of China, Chengdu 610072, China; seay_angany@sina.com (Y.Y.); 18180585806@163.com (M.S.); 4 Department of Neurosurgery, Sichuan Academy of Medical Sciences & Sichuan Provincial People’s Hospital, School of Medicine, University of Electronic Science and Technology of China, Chengdu 610072, China; jchonggui@163.com

**Keywords:** stroke-associated pneumonia, neurorehabilitation, nasogastric tube, nutrition

## Abstract

**Highlights:**

**What are the main findings?**

- Admission inflammatory markers (WBC, CRP, fibrinogen) and low nutritional status (especially low albumin) are independently associated with a higher risk of stroke-associated pneumonia (SAP).
- SAP is strongly associated with poorer short-term functional recovery and increased healthcare burden (longer hospital stay and higher costs).

**What are the implications of the main findings?**

- Routine admission biomarkers can support early risk stratification of SAP, helping identify high-risk stroke patients for closer monitoring and preventive strategies.
- Targeted interventions (e.g., nutritional assessment/support, dysphagia management) should be explored in prospective studies to determine whether modifying these factors can improve outcomes.

**Abstract:**

Background: Stroke-associated pneumonia (SAP) is a common complication after acute ischemic stroke and contributes to worse recovery and greater resource use. Nutritional and inflammatory dysregulation have been implicated in both SAP susceptibility and adverse prognosis. Objective: To examine whether admission inflammatory and nutritional markers are associated with the development of SAP and with short-term functional prognosis. Methods: We performed a retrospective single-centre cohort study of consecutive patients with acute ischemic stroke admitted between 1 January 2015 and 31 December 2024 (N=303;SAPn=108,non−SAPn=195). Admission laboratory indices (albumin, CRP, fibrinogen, WBC, PCT, and prealbumin) in the first 24 h and clinical variables were analysed. Multivariable logistic regression identified factors independently associated with SAP; the relationship between SAP and early functional recovery was assessed in adjusted outcome models. A nomogram integrating key predictors was developed and its apparent discrimination is reported. Results: SAP occurred in 35.6% of patients. Factors independently associated with SAP included nasogastric tube placement (OR: 7.02, 95% CI: 3.50–14.62), venous thromboembolism (OR: 3.20, 95% CI: 1.62–6.31), cognitive impairment (OR: 2.90, 95% CI: 1.32–6.36), and elevated inflammatory markers (WBC OR: 1.52, 95% CI: 1.28–1.80; fibrinogen OR: 1.37, 95% CI: 1.02–1.84; CRP OR: 1.01, 95% CI: 1.00–1.03). Higher admission serum albumin was associated with lower odds of SAP (OR: 0.92, 95% CI: 0.86–0.98). The nomogram showed strong apparent discrimination (AUC: 0.90, 95% CI: 0.86–0.94). After multivariable adjustment, SAP remained associated with poorer short-term functional improvement (adjusted OR: 6.99, 95% CI: 3.05–17.54) and greater healthcare utilization (median length of stay: 39.6 vs. 30.6 days; median cost: USD 12,836 vs. 6585). Conclusion: In this retrospective cohort, admission markers of nutritional depletion and inflammatory activation were associated not only with increased likelihood of SAP, but also with adverse early functional outcomes. These association-based findings support early risk stratification using routine admission markers; prospective studies and external validation are required before clinical implementation.

## 1. Introduction

Stroke remains a leading cause of mortality and long-term disability worldwide. Among patients hospitalized with acute stroke, infectious complications are common and contribute substantially to worse outcomes; in particular, stroke-associated pneumonia (SAP) is one of the most common post-stroke infections and is consistently associated with prolonged hospitalization, increased mortality, and poorer functional recovery [1].

In pneumonia populations more broadly, systemic inflammatory activation and impaired nutritional status have been associated with disease severity and prognosis. Several recent cohort studies and systematic investigations indicate that biomarkers reflecting inflammatory response and nutritional reserve—including C-reactive protein (CRP), leukocyte-derived indices, fibrinogen, and serum albumin, as well as composite immune-nutritional scores such as the prognostic nutritional index (PNI)—are associated with severity and short-term mortality in community-acquired and hospital-acquired pneumonia [2,3].

Although stroke-specific mechanisms (for example, dysphagia, reduced consciousness, and stroke-induced immune alterations) increase the risk of pulmonary infection, the biological mediators that determine susceptibility and prognosis in SAP substantially overlap with those described in non-stroke pneumonia cohorts; composite inflammation–nutrition indices (e.g., CRP/albumin ratio, CAR [4,5], and PNI [6]) have recently been explored both as predictors of pneumonia severity and as prognostic markers after respiratory infection.

Given these considerations, it is important to characterize whether admission inflammatory and nutritional biomarkers are associated with the occurrence of stroke-associated pneumonia (SAP) and whether they are related to subsequent post-stroke functional outcomes. The present study was therefore designed to evaluate these associations in order to improve early clinical risk stratification in patients with acute ischemic stroke. Consistent with this focus, our analyses were restricted to admission (baseline) nutritional status rather than to in-hospital nutritional interventions or serial post-admission measurements.

## 2. Methods

### 2.1. Data Collection and Cohort Stratification

This retrospective cohort study included consecutive patients with acute ischemic stroke admitted to a single tertiary center between 1 January 2015 and 31 December 2024. Patients were classified as having stroke-associated pneumonia (SAP) (n=108) or no SAP (n=195). SAP was defined as a lower respiratory tract infection occurring within 7 days after stroke onset based on clinical features, inflammatory markers, and radiographic findings [7]. Demographic and clinical variables, including age, sex, body mass index (BMI), vascular risk factors, chronic obstructive pulmonary disease (COPD), and venous thromboembolism (VTE), were extracted from electronic medical records.

Laboratory indices obtained at hospital admission (within 24 h) were pre-specified as exposures of interest and included white blood cell (WBC) count, neutrophil count, hemoglobin, albumin, prealbumin, procalcitonin (PCT), C-reactive protein (CRP), and fibrinogen. By study design, serial post-admission albumin measurements and systematic records of in-hospital nutritional or glycemic interventions were not collected and were therefore unavailable for analysis. Likewise, data on dental status, routine proton pump inhibitor (PPI) use, smoking history, admission D-dimer levels, and detailed diabetes characteristics (duration, HbA1c, and treatment) were inconsistently recorded and were excluded from the analysis.

Admission National Institutes of Health Stroke Scale (NIHSS) scores [8] and detailed lesion metrics were incompletely documented and were therefore not included in the primary multivariable models, which may allow for residual confounding due to stroke severity. To partially address this limitation, we adjusted for available clinical proxies of neurological impairment, including dysphagia (documented bedside swallowing assessment), impaired consciousness, nasogastric tube placement, and post-stroke cognitive impairment (CI). CI was defined based on medical record documentation, including a recorded diagnosis of dementia, a Mini-Mental State Examination (MMSE) score < 24 when available [9], or physician-documented confusion or disorientation. Nasogastric tube use was treated as a proxy for severe dysphagia or neurological compromise rather than as a causal exposure.

### 2.2. Statistical Analysis and Model Construction

Continuous variables were tested for normality. Due to potential outliers in laboratory variables, median and interquartile range (IQR) are reported for these measures. Inter-group differences were assessed via Mann–Whitney U test or Student’s *t*-test as appropriate. Categorical variables were expressed as frequencies (percentages) and compared using the Chi-square test or Fisher’s exact test.

To identify factors independently associated with SAP, variables exhibiting statistical significance (*p* < 0.05) in the univariate analysis were entered into a multivariate binary logistic regression model. Results were reported as odds ratios (ORs) with 95% confidence intervals (CIs). Subsequently, a prognostic nomogram was constructed based on the identified independent predictors to provide a visual probabilistic tool for clinicians. The discriminative performance of the model was quantified using the Receiver Operating Characteristic (ROC) curve and the Area Under the Curve (AUC). Model calibration was evaluated via calibration plots comparing predicted versus observed probabilities, and clinical utility was assessed using Decision Curve Analysis (DCA) to estimate the net benefit across threshold probabilities.

### 2.3. Outcome Assessment

The prognostic impact of SAP was evaluated through three distinct dimensions: functional recovery (quantified by the magnitude of improvement in the modified Rankin Scale, mRS), economic burden (hospitalization costs), and resource utilization (length of stay). To determine the independent association of SAP with neurological outcomes, a separate multivariate logistic regression analysis was performed, adjusting for confounding variables, with poor prognosis defined as an mRS improvement of <2 points.

Data analysis was conducted using IBM SPSS Statistics for Windows, version 30.0 (IBM Corp., Armonk, NY, USA), with packages “rms” for regression modeling, “pROC” for ROC analysis, and “rmda” for decision curve analysis. A two-tailed *p* < 0.05 was considered statistically significant.

## 3. Results

### 3.1. Baseline Characteristics and Nutritional-Inflammatory Profile

The final analysis included 303 patients, of whom, 108 (35.6%) developed stroke-associated pneumonia (SAP) within 7 days after stroke onset. Baseline characteristics of patients with and without SAP are summarized in Table 1. Age, sex, and body mass index (BMI) were comparable between the groups (*p* > 0.05). However, the SAP group showed a significantly higher prevalence of certain comorbidities and invasive interventions. In particular, venous thromboembolism (VTE) and nasogastric tube placement were more common in patients with SAP than in those without SAP (32.4% vs. 13.8% and 63.9% vs. 15.4%, respectively; *p* < 0.001).

Baseline characteristics stratified by functional outcome (poor vs. good outcome) are presented in Table 2. Table 1 and Table 2 present baseline characteristics stratified by SAP status and functional outcome, respectively.

Regarding nutritional and inflammatory indices, patients with SAP showed higher inflammatory marker levels and lower nutritional markers. White blood cell (WBC) counts were higher in the SAP group (median [IQR]: 8.72 [7.32–10.78] vs. 6.83 [5.89–8.12] × 10^9^/L, *p* < 0.001), as were C-reactive protein (CRP) levels (median [IQR]: 12.35 [4.82–28.60] vs. 2.85 [1.20–6.45] mg/L, *p* < 0.001). In contrast, nutritional indicators were lower in patients with SAP, including serum albumin (median [IQR]: 38.20 [34.50–41.00] vs. 40.50 [37.20–43.10] g/L, *p* < 0.001) and prealbumin (median [IQR]: 198.5 [168.0–232.0] vs. 212.0 [180.0–245.0] mg/L, *p* = 0.044). Because several laboratory variables showed non-normal distributions, continuous variables are reported as medians and interquartile ranges (IQRs).

### 3.2. Factors Independently Associated with SAP

**Figure 1:**
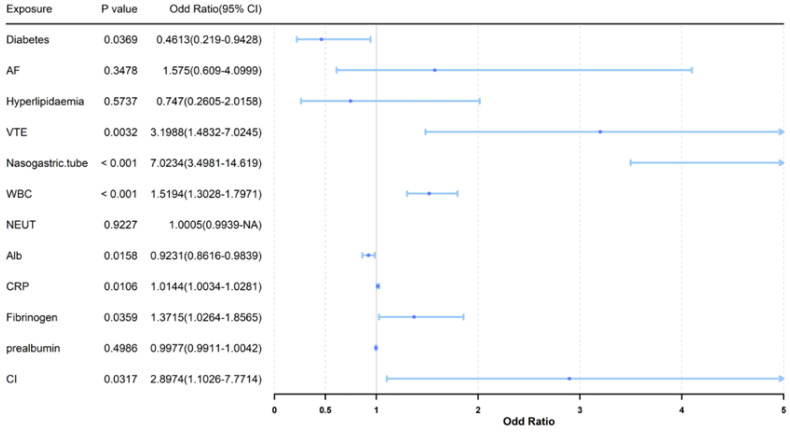
Logistic regression analysis of factors associated with stroke-associated pneumonia.

Multivariable logistic regression identified several factors independently associated with SAP (Figure 1). Variables entered into the model were selected based on clinical relevance and variables with *p* < 0.10 in univariate analysis. Nasogastric tube placement showed the strongest association with SAP (OR = 7.02, 95% CI: 3.50–14.62, *p* < 0.001). Other variables independently associated with SAP included VTE (OR = 3.20, 95% CI: 1.62–6.31), cognitive impairment (OR = 2.90, 95% CI: 1.32–6.36), WBC count (OR = 1.52, 95% CI: 1.28–1.80, *p* < 0.001), and fibrinogen (OR = 1.37, 95% CI: 1.02–1.84, *p* = 0.036).

Serum albumin levels were inversely associated with SAP (OR = 0.92, 95% CI: 0.86–0.98, *p* = 0.016). Diabetes was also included as a binary comorbidity variable and showed an inverse association with SAP (OR = 0.46, 95% CI: 0.24–0.88).

### 3.3. Nomogram Construction and Validation

**Figure 2:**
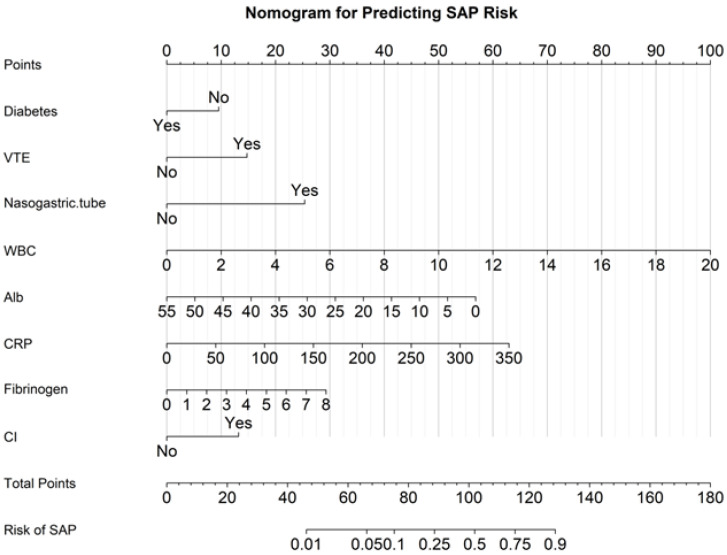
Nomogram for predicting stroke-associated pneumonia.

**Figure 3:**
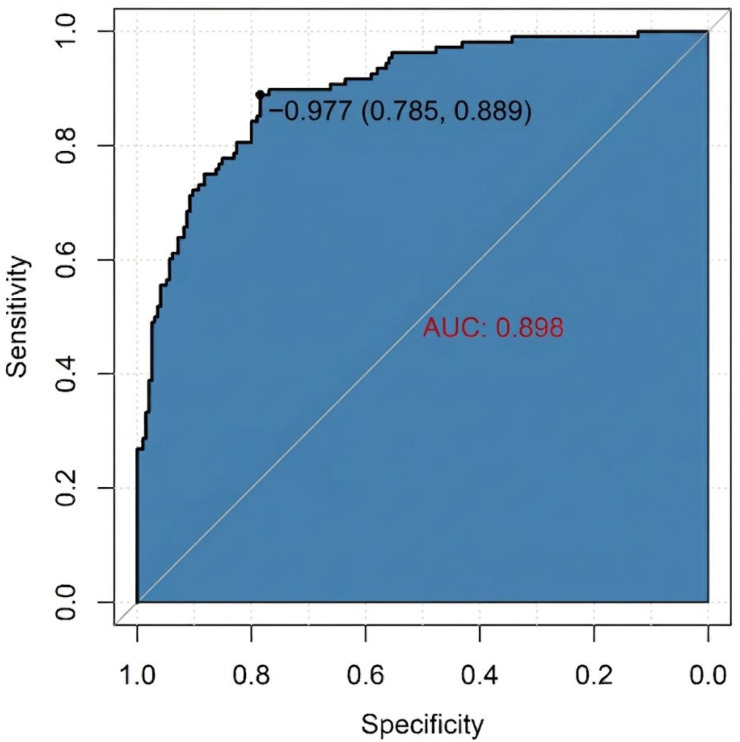
ROC curve of the nomogram for predicting stroke-associated pneumonia.

A nomogram incorporating eight predictors (diabetes, VTE, nasogastric tube placement, WBC count, albumin, CRP, fibrinogen, and cognitive impairment) was developed to estimate the probability of SAP (Figure 2). The model demonstrated good discrimination, with an area under the receiver operating characteristic curve (AUC) of 0.90 (95% CI: 0.86–0.94) (Figure 3). The nomogram was developed and evaluated within the same cohort.

**Figure 4:**
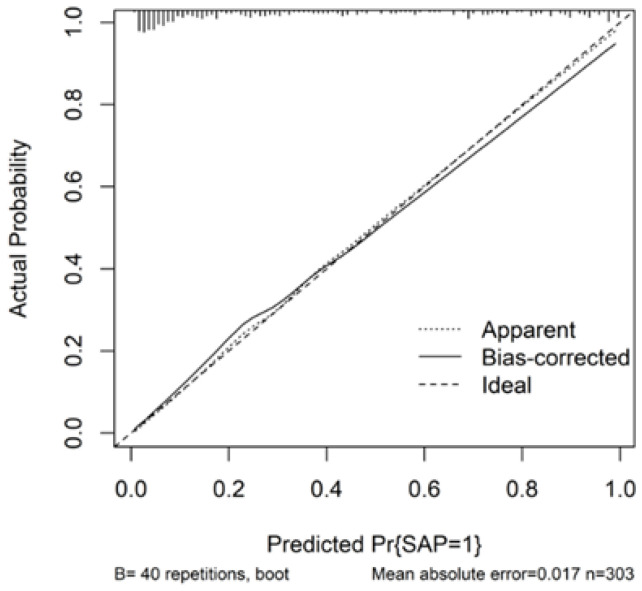
Calibration curve of the nomogram for predicting stroke-associated pneumonia.

**Figure 5:**
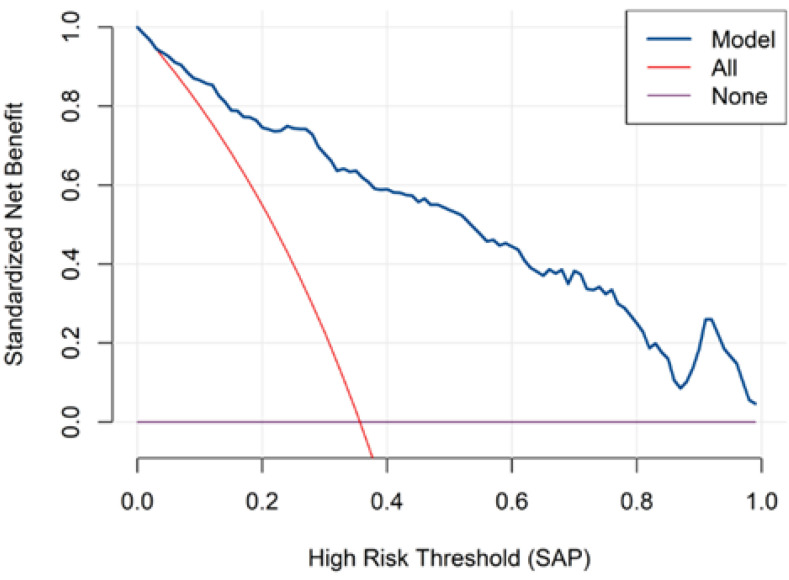
Decision curve analysis evaluating the clinical utility of the nomogram.

Calibration analysis showed reasonable agreement between predicted and observed probabilities (Figure 4). Decision curve analysis suggested potential clinical utility of the model across a range of threshold probabilities (Figure 5).

### 3.4. SAP Association with Recovery Outcomes

SAP was significantly associated with post-stroke functional outcomes. Patients with SAP showed smaller improvements in mRS scores than those without SAP, as reflected by smaller changes in modified Rankin Scale (mRS) scores (*p* < 0.001) (Table 2).

**Figure 6:**
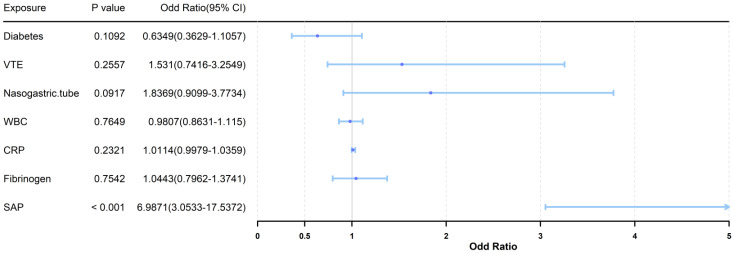
Forest plot of multivariable logistic regression analysis for ischemic stroke prognosis.

In multivariable analysis adjusted for potential confounders, including age, baseline mRS score, and available clinical indicators of neurological impairment, SAP remained independently associated with poor functional outcome (defined as mRS improvement < 2) (OR = 6.99, 95% CI: 3.05–17.54, *p* < 0.001) (Figure 6).

SAP was also associated with greater healthcare resource utilization. Patients with SAP had higher hospitalization costs (USD 12,836 ± 8079 vs. USD 6585 ± 3983, *p* < 0.001) and longer hospital stays (39.55 ± 19.55 days vs. 30.56 ± 13.77 days, *p* < 0.001) (Table 3). Costs and length of stay are reported as mean ± SD.

## 4. Discussions

Stroke-associated pneumonia (SAP) remains a common complication after acute ischemic stroke. In this cohort, SAP occurred in 35.6% of patients, a prevalence comparable to prior studies [10,11,12], and was associated with worse functional recovery and greater healthcare utilization [13,14].

Consistent with previous work, SAP in our study was linked primarily to acute physiological and functional factors rather than baseline vascular comorbidity. Markers of systemic inflammation measured at admission—white blood cell count (OR: 1.52, 95% CI: 1.28–1.80), fibrinogen (OR: 1.37, 95% CI: 1.02–1.84) and C-reactive protein (OR: 1.01, 95% CI: 1.00–1.03)—remained independently associated with SAP after adjustment. Early elevations in these markers may, however, partly reflect incipient infection or aspiration, and reverse causality cannot be excluded; these findings nonetheless support the role of post-stroke inflammatory activation in SAP susceptibility [7,10,11,15,16].

Lower admission serum albumin was independently associated with higher odds of SAP (OR: 0.92, 95% CI: 0.86–0.98). This aligns with literature linking malnutrition and hypoalbuminaemia to greater infection susceptibility after stroke [17] and with meta-analytic evidence that low admission albumin predicts greater pneumonia severity and worse outcomes [18]. Albumin should be viewed primarily as a marker of physiological/nutritional vulnerability rather than as proof of a direct protective mechanism; composite immune-nutritional indices that include albumin (for example, prognostic nutritional index and CRP–albumin ratio) have shown reproducible associations with severity and short-term mortality in pneumonia and stroke cohorts [19,20]. Because low albumin at admission often reflects pre-existing malnutrition or chronic illness rather than an immediate post-stroke catabolic fall, serial measurements are required to distinguish chronic from acute hypoalbuminaemia [19]. Taken together, these observations support routine early nutritional assessment on admission and consideration of targeted nutritional support for patients with low albumin, although randomized evidence that supplementation reduces pneumonia incidence or hard clinical endpoints remains limited [21].

Neurological impairment and dysphagia-related variables were strongly associated with SAP; nasogastric tube placement had the largest adjusted association (OR: 7.02, 95% CI: 3.50–14.62). We interpret nasogastric tube use primarily as a proxy for severe dysphagia, impaired consciousness and compromised airway protection, rather than as a modifiable causal exposure. Prior studies have linked dysphagia and impaired consciousness with increased aspiration and pneumonia risk after stroke [22,23,24]. Cognitive impairment was also independently associated with SAP (OR: 2.90, 95% CI: 1.32–6.36), an association that may reflect reduced airway clearance, impaired adherence to swallowing precautions, or delayed mobilization, although these mechanisms remain speculative in our datasets [13,25].

Diabetes has consistently been associated with increased incidence of community-acquired pneumonia in observational studies and meta-analyses; for example, a systematic review including more than 14 million participants reported a pooled relative risk of approximately 1.64 for CAP among individuals with type 2 diabetes compared with those without diabetes [26]. However, accumulating evidence suggests that acute metabolic status at presentation, particularly admission hyperglycaemia, may be more strongly associated with adverse pneumonia outcomes than the binary diagnosis of diabetes itself. A recent meta-analysis including more than 34,000 patients demonstrated that elevated admission glucose was significantly associated with increased short-term mortality (pooled OR ≈ 2.67) and higher rates of ICU admission among patients hospitalized with pneumonia [27]. While these CAP data underline the importance of metabolic control in pneumonia generally, stroke-associated pneumonia (SAP) arises at the intersection of systemic metabolic status and stroke-specific vulnerabilities (for example, dysphagia, impaired consciousness, and stroke-induced immunological alterations), so metabolic factors may interact with—rather than replace—neurological determinants of SAP risk and prognosis.

In our retrospective dataset, diabetes was recorded as a binary comorbidity only and information on duration, HbA1c or inpatient glycaemic management was not consistently available; differential inpatient monitoring or treatment is therefore a plausible but untestable explanation for the lower diabetes prevalence observed in some subgroups. The study was designed to evaluate admission inflammatory–nutritional markers and did not systematically capture in-hospital glycaemic or nutritional interventions; this limitation may allow for residual confounding but does not alter the study’s primary focus or the reported admission-marker associations with SAP.

Venous thromboembolism was also associated with SAP (OR: 3.20, 95% CI: 1.62–6.31), which may reflect clustering of immobility, systemic inflammation and overall illness severity rather than a direct causal link between thrombosis and pneumonia [10,11]. Several classical vascular risk factors (for example, hypertension and atrial fibrillation) did not retain independent predictive value in adjusted models, suggesting that acute neurological dysfunction, dysphagia, inflammatory activation and nutritional depletion exert a more immediate influence on early SAP risk than chronic comorbidity burden [7,10,22,28].

We constructed a nomogram integrating inflammatory, nutritional, neurological and procedural variables that demonstrated good discrimination in this cohort (AUC: 0.90, 95% CI: 0.86–0.94). This model emphasizes factors that are directly relevant to rehabilitation-oriented management strategies [7,13,15]. Because the model was developed and evaluated in the same retrospective sample without external validation or internal resampling, its performance may be optimistic and susceptible to overfitting; calibration and independent validation are required before clinical implementation.

Finally, SAP remained associated with poorer functional outcomes after multivariable adjustment (mRS improvement < 2 points OR: 6.99, 95% CI: 3.05–17.54, *p* < 0.001), and SAP patients experienced longer hospital stays (39.5 vs. 30.5 days) and higher costs (USD 12,836 vs. 6585). These associations should be interpreted in the context of overall illness severity and complications (reverse causality is possible), and SAP should be considered a marker of a broader severity-driven vulnerability profile rather than a sole mediator of poor recovery [13,15].

In summary, our findings reinforce the need for early identification of high-risk patients using readily available admission markers and for prospective studies that evaluate targeted interventions (nutritional support, dysphagia management, early mobilisation and glycaemic control) to determine whether modifying these factors can reduce SAP incidence and improve recovery.

## 5. Limitations

Despite these limitations, the study leverages a real-world acute ischemic stroke cohort with systematically collected admission biomarkers and clinical data, providing hypothesis-generating evidence for early risk stratification of SAP.

Study design and generalizability. This retrospective single-center study cannot establish causality, and the SAP and control groups are unevenly sized, which may introduce selection bias.

Timing and completeness of measurements. Nutritional and inflammatory markers were assessed at admission only; serial biomarker data and systematic records of in-hospital nutritional or glycaemic interventions were not collected by study design and therefore could not be evaluated as potential modifiers of SAP risk. Early elevations in inflammatory markers may partly reflect incipient infection or aspiration rather than pre-existing risk.

Neurological severity and residual confounding. Admission NIHSS and detailed lesion metrics were incompletely documented (2015–2024); we therefore used chart-documented post-stroke cognitive impairment and clinical proxies (dysphagia, impaired consciousness, and nasogastric tube placement) to adjust for neurological compromise. These proxies are imperfect, formal validation across the full cohort was not feasible, and residual confounding by unmeasured stroke severity cannot be excluded.

Modeling and missing covariates. Some predictors (nasogastric tube use, inflammatory markers, and CI) may reflect underlying severity or early infection rather than independent causal effects. The nomogram was developed and tested in the same cohort without external validation (and without internal resampling), so overfitting may have inflated apparent performance. Key covariates—smoking history, admission D-dimer, and detailed diabetes phenotyping (duration, HbA1c, and treatment)—were not uniformly recorded and were excluded; this may have caused residual confounding.

These constraints argue for cautious interpretation and highlight the need for prospective, multicentre validation with standardized severity metrics and systematic collection of metabolic and nutritional interventions.

## 6. Conclusions

In this single-centre retrospective cohort, stroke-associated pneumonia (SAP) was associated with admission markers of nutritional depletion, systemic inflammation, and neurological impairment, and SAP was independently associated with poorer short-term functional recovery and greater healthcare resource use. These findings are observational and should not be interpreted as causal given the potential residual confounding and the retrospective design. Nevertheless, they support the value of early risk stratification using readily available admission markers to identify patients who might benefit from targeted, hypothesis-driven interventions (for example, nutritional assessment, dysphagia management, and optimized glycaemic care), which require confirmation in prospective studies. Finally, external validation of the proposed predictive model and prospective studies with standardized stroke-severity metrics and serial biomarker measurements are needed before clinical implementation.

## Figures and Tables

**Table 1:** Baseline characteristics of patients stratified by stroke-associated pneumonia (SAP).

Characteristic	Non-SAP Group (*n* = 195)	SAP Group (*n* = 108)	*p*-Value
Demographic Variables			
Age, years	65.44 ± 13.67	67.05 ± 13.52	0.325
Male sex, *n* (%)	127 (65.1)	71 (65.7)	1.000
BMI, kg/m^2^	23.96 ± 3.51	23.70 ± 3.64	0.536
Vascular Risk Factors, *n* (%)			
Hypertension	149 (76.4)	79 (73.1)	0.623
Diabetes	81 (41.5)	28 (25.9)	0.010
Atrial fibrillation	16 (8.2)	24 (22.2)	0.001
Hyperlipidemia	43 (22.1)	12 (11.1)	0.027
Comorbidities, *n* (%)			
COPD	1 (0.5)	2 (1.9)	0.602
Venous thromboembolism (VTE)	27 (13.8)	35 (32.4)	<0.001
Procedure-related Variables, *n* (%)			
Nasogastric tube placement	30 (15.4)	69 (63.9)	<0.001
Laboratory Variables			
WBC count, ×10^9^/L [M (IQR)]	6.83 (5.89–8.12)	8.72 (7.32–10.78)	<0.001
Neutrophil count, ×10^9^/L [M (IQR)]	4.49 (3.47–6.26)	6.56 (4.61–8.42)	0.004
Hemoglobin, g/L [M (IQR)]	139 (127–150)	133 (120–144)	0.456
Albumin, g/L [M (IQR)]	40.50 (37.20–43.10)	38.20 (34.50–41.00)	<0.001
Procalcitonin, μg/L [M (IQR)]	0.18 (0.10–0.28)	0.45 (0.20–1.20)	0.129
CRP, mg/L [M (IQR)]	2.85 (1.20–6.45)	12.35 (4.82–28.60)	<0.001
Fibrinogen, g/L [M (IQR)]	2.82 (2.37–3.27)	3.10 (2.48–3.86)	<0.001
Prealbumin, mg/L [M (IQR)]	212.0 (180.0–245.0)	198.5 (168.0–232.0)	0.044
Neurological Variables, *n* (%)			
Cognitive impairment (CI)	14 (7.2)	18 (16.7)	0.017

Abbreviations: BMI, body mass index; COPD, chronic obstructive pulmonary disease; WBC, white blood cell; CRP, C-reactive protein; M, median; IQR, interquartile range. Note: Due to potential outliers in laboratory variables, median and interquartile range (IQR) are reported instead of mean and standard deviation.

**Table 2:** Baseline characteristics stratified by functional outcomes after ischemic stroke.

Characteristic	Poor Functional Outcome (mRS Improvement < 2) (*n* = 195)	Good Functional Outcome (mRS Improvement ≥ 2) (*n* = 108)	*p*-Value
Demographic Variables			
Age, years	65.84 ± 13.41	66.31 ± 14.01	0.774
Male sex, *n* (%)	125 (65.1)	73 (65.8)	1.000
BMI, kg/m^2^	23.63 ± 3.61	24.28 ± 3.42	0.128
Vascular Risk Factors, *n* (%)			
Hypertension	142 (74.0)	86 (77.5)	0.585
Diabetes	59 (30.7)	50 (45.0)	0.017
Atrial fibrillation	27 (14.1)	13 (11.7)	0.685
Hyperlipidemia	34 (17.7)	21 (18.9)	0.913
Comorbidities, *n* (%)			
COPD	2 (1.0)	1 (0.9)	1.000
Venous thromboembolism (VTE)	48 (25.0)	14 (12.6)	0.015
Procedure-related Variables, *n* (%)			
Nasogastric tube placement	83 (43.2)	16 (14.4)	<0.001
Laboratory Variables			
WBC count, ×10^9^/L [M (IQR)]	8.35 (6.80–10.20)	7.36 (6.10–8.60)	0.001
Neutrophil count, ×10^9^/L [M (IQR)]	6.20 (4.80–8.10)	5.10 (4.00–6.50)	0.152
Hemoglobin, g/L [M (IQR)]	142 (130–154)	138 (126–150)	0.566
Albumin, g/L [M (IQR)]	38.84 (35.60–42.10)	39.48 (36.20–43.50)	0.279
Procalcitonin, μg/L [M (IQR)]	0.33 (0.15–0.60)	0.20 (0.10–0.40)	0.230
CRP, mg/L [M (IQR)]	18.91 (6.50–42.50)	4.48 (1.80–9.20)	0.001
Fibrinogen, g/L [M (IQR)]	3.65 (2.90–4.40)	3.27 (2.60–3.90)	0.008
Prealbumin, mg/L [M (IQR)]	207.83 (175.0–240.0)	214.41 (182.0–248.0)	0.329
Neurological Variables, *n* (%)			
Cognitive impairment (CI)	23 (12.0)	9 (8.1)	0.388
Outcome Variables, *n* (%)			
Stroke-associated pneumonia (SAP)	99 (51.6)	9 (8.1)	<0.001

Abbreviations: BMI, body mass index; COPD, chronic obstructive pulmonary disease; WBC, white blood cell; CRP, C-reactive protein; mRS, modified Rankin Scale; M, median; IQR, interquartile range. Note: Due to potential outliers in laboratory variables, median and interquartile range (IQR) are reported instead of mean and standard deviation. Poor functional outcome was defined as mRS improvement < 2 points; good functional outcome was defined as mRS improvement ≥ 2 points.

**Table 3:** Comparison of hospitalization costs and length of stay between patients with and without stroke-associated pneumonia.

Variable	Non-SAP Group (*n* = 195)	SAP Group (*n* = 108)	t-Statistic	*p*-Value
Hospitalization costs, USD	6585 ± 3983	12,836 ± 8079	7.5515	<0.001
Length of stay, days	30.56 ± 13.77	39.55 ± 19.55	4.2309	<0.001

Note: Hospitalization costs were converted from Chinese Yuan (RMB) to US Dollars (USD) using an exchange rate of 1 USD = 7.16 RMB. Values are presented as mean ± standard deviation.

## Data Availability

Data is contained within the article.

## Abbreviations

The following abbreviations are used in this manuscript:

SAP	Stroke-associated pneumonia
BMI	Body mass index
COPD	Chronic obstructive pulmonary disease
VTE	Venous thromboembolism
WBC	White blood cell
NEUT	Neutrophil
Hb	Hemoglobin
Alb	Albumin
PCT	Procalcitonin
CI	Cognitive impairment
CRP	C-reactive protein
OR	Odds ratio
ROC	Receiver operating characteristic
DCA	Decision curve analysis
AUC	Area under the curve
95% CI	95% confidence interval
